# Effects of an Intensive Gait Training Rehabilitation Protocol After Single Event Multilevel Surgery in Cerebral Palsy: Activ’Marche

**DOI:** 10.3390/jcm15197398

**Published:** 2026-09-23

**Authors:** Farah Ayoubi, Karim Soualem, Magali Contrecivil, Florian Breton, Santhis Jabely Duchesne, Elodie Hinnekens, Lionel Lejeune, Sandrine Payan-Terral, Anne-Laure Guinet, Constance Amelon-Petit, Michel Bams, Eric Desailly

**Affiliations:** 1 Pôle Recherche & Innovation, Fondation Ellen Poidatz, Saint Fargeau Ponthierry, 77310 Saint-Fargeau-Ponthierry, France; farah.ayoubi@fondationpoidatz.com (F.A.);; 2 Centre de Rééducation Fonctionnelle, Fondation Ellen Poidatz, Saint Fargeau Ponthierry, 77310 Saint-Fargeau-Ponthierry, France

**Keywords:** intensive rehabilitation, single event multilevel surgery, cerebral palsy

## Abstract

**Objective:** Functional gait parameters of children with cerebral palsy (CP) are not systematically improved after conventional rehabilitation post-single-event multilevel surgery (SEMLS). To enhance functional outcomes in patients one year post-surgery, a new intensive rehabilitation phase called “Activ’Marche” was added to the usual protocol. This late intensive phase was expected to improve gait function and motor performance. **Methods**: Design: Quasi-experimental pre–post intervention study with 3-month follow-up. Setting: In-care rehabilitation center followed by home-based telerehabilitation. Participants: 23 adolescents with CP (age 12–19 years), classified as Gross Motor Function Classification System (GMFCS) levels I-II, one year after SEMLS. Intervention: 6-week intensive rehabilitation program consisting of 4 weeks of full-time in-care rehabilitation followed by 2 weeks of home-based telerehabilitation. The program combined high-intensity interval training, muscle strengthening, and functional gait training. Main measures: Activity capacity was assessed by 6-Minute Walk test (6MWT), 10-Meter Walk test (10MWT) and Gait Deviation Index (GDI) computation using clinical gait analysis; activity performance was measured by Gross Motor Function Measure (GMFM) D/E score, and one week of actimetric recording, which also partially reflects participation. Quality of life was assessed by the Pediatric Quality of Life Inventory (PedsQL). **Results:** Significant improvements were observed in the 6MWT (+13%, *p* = 0.008), GMFM-66 dimensions D (+6.7%, *p* = 0.007) and E (+8.5%, *p* < 0.001), and 10MWT (+14,2%, *p* = 0.002), with gains maintained or enhanced at follow-up. **Conclusions:** This new intensive rehabilitation protocol significantly improves overall motor function and walking performance in adolescents with CP following SEMLS without affecting gait quality or quality of life.

## 1. Introduction

Cerebral palsy (CP) is defined as a “group of permanent disorders of the development of movement and posture, causing activity limitation” [1]. Children with CP often develop altered gait patterns leading to mobility restrictions. Single-event multilevel surgery (SEMLS) is widely used to correct musculoskeletal deformities affecting gait by addressing multiple orthopedic impairments during a single operative session. SEMLS followed by postoperative rehabilitation is effective for children with CP, helping them to improve passive range of motion, gait kinematics, overall gait index and energy efficiency [2,3,4,5,6].

Postoperative rehabilitation following SEMLS is long and complex, requiring an average of five months of hospitalization, structured into several phases. Guinet et al. proposed a Five-Step Framework to describe rehabilitation after SEMLS [7]. Gait training is introduced in the third phase (weeks 2–6 post-surgery). The fourth step (week 7 to month 6 post-surgery) represents an intensification period in the rehabilitation process. The final step extends up to two years postoperatively, focusing on maintaining muscle strength and range of motion.

While SEMLS effectively addresses orthopedic deformities, current postoperative rehabilitation protocols remain highly heterogeneous, with no evidence-based consensus regarding content, intensity, dosage, or progression [5,8]. This variability limits the reproducibility of rehabilitation programs and may contribute to inconsistent functional outcomes after SEMLS.

Two main factors may contribute to this limitation. First, rehabilitation organization is often suboptimal: the fourth and fifth steps of rehabilitation after SEMLS are often shortened or skipped due to the prolonged duration of the initial inpatient phase, decreased patient adherence and reduced rehabilitation intensity after the return home.

Second, rehabilitation content may lack relevance. Despite the well-established importance of postoperative rehabilitation, there is a lack of detailed information in the literature regarding the type, intensity and frequency of outpatient rehabilitation, particularly in the later phases. To enhance motor recovery, current motor learning theories recommend task-specific, variable and high-intensity practice [9]. Three types of training have been identified as effective in improving functional gait performance in patients with CP: functional gait training, strength training, and high-intensity interval training (HIIT). Previous studies have demonstrated that functional gait training, including overground or treadmill gait training, with or without body weight support, can enhance walking speed, endurance, and overall motor function when performed for 30 min sessions, twice per week, over a period of 7 weeks [10,11,12,13,14]. Moreover, strength training through circuit-based exercises, combining bodyweight and machine-based resistance training, conducted in 60 min sessions, three times per week, over five weeks, has been shown to enhance walking speed and overall motor function [15,16,17]. Zwinkels et al. demonstrated that HIIT training exercises performed for 20 to 25 min twice per week over an eight-week period enhance both aerobic and anaerobic performance [18]. However, these approaches have predominantly been investigated as separate interventions, and little is known about the potential benefits of integrating them into a single, intensive multimodal rehabilitation program. Furthermore, there is currently no consensus regarding the optimal dosage and intensity to be prescribed for children with CP. Combining these complementary approaches could provide synergistic benefits, as improvements in one domain might facilitate progress in another and promote general engagement in motor practice, while enabling exploration of an adjusted dosage.

Moreover, there is limited evidence regarding the effectiveness of introducing a new period of intensive rehabilitation approximately one year after SEMLS, when patients have generally completed the conventional postoperative rehabilitation pathway and rehabilitation intensity may have substantially decreased.

To address these limitations and promote significant functional improvement, two strategies can be considered. The first strategy is organizational, and introduces a new inpatient rehabilitation period one year after surgery, referred to as phase 4 bis. Phase 4 bis represents a secondary intensification period modeled after the original fourth step of the Guinet et al. framework. This additional phase is specifically designed to re-engage patients and sustain progress through more stimulating and context-relevant practices. The second strategy focuses on the qualitative and scientific foundations of the rehabilitation protocols, by carefully selecting and integrating evidence-based approaches within the program.

The aim of this study was to assess the effects of introducing an additional intensive multimodal rehabilitation phase one year after SEMLS on gait performance in children and adolescents with CP. All participants had previously undergone SEMLS and completed standard postoperative rehabilitation. The investigated intervention consisted exclusively of a combined intensive multimodal therapy program (“Activ’Marche”) integrating functional gait training, strength training, and high-intensity interval training (HIIT), followed by a home exercise program. We hypothesized that adding this late intensive phase would improve functional gait parameters and motor performance.

## 2. Materials and Methods

### 2.1. Study Design

This study was a quasi-experimental study with pre- and post-intervention measurements. Outcome measures were assessed at baseline, immediately after the intervention and at a three-month follow-up. This study received agreement from the appropriate ethics committee in France (Comité de Protection des Personnes Nord-Ouest II—Number 2021-A00921-40).

### 2.2. Participants

The inclusion criteria identified children, adolescents and young adults with CP who underwent a SEMLS over one year beforehand, aged between 10 to 20 years, classified I to III according to the Gross Motor Function Classification System (GMFCS), and able to understand the instructions and to cooperate. Exclusion criteria were the presence of injuries or pain in the lower limbs preventing intensive training, administration of botulinum toxin or surgical intervention within the six months prior to study inclusion, and participation in a structured rehabilitation program within the six months prior to study inclusion.

### 2.3. Outcome Measures

Outcomes of the study were selected to cover the levels of the International Classification of Functioning, Disability and Health: Children & Youth version (ICF-CY) [19]. Activity capacity was assessed by the 6-Minute Walk test (6MWT), the 10-Meter Walk test, and GDI computation using clinical gait analysis; activity performance was evaluated by the Gross Motor Function Measure-66 (GMFM-66) and by one week of actimetric recording, which also partially represented participation. Quality of life was assessed using PedSQL.

### 2.4. Primary Outcomes

The 6MWT, as primary outcome, measures the submaximal level of an individual’s functional capacities during a spontaneous walking exercise [20]. Participants were instructed to walk as far as possible for six minutes, and the total distance covered was recorded. The 6MWT is a widely used and reliable measure of functional walking capacity in children with CP [21].

### 2.5. Secondary Outcomes

#### 2.5.1. Activity Capacity Assessment

Clinical gait analysis was performed using a fifteen-camera VICON system (8 MX 20, 5 T 40, 2 T 160) (PluginGait marker set, VICON, Oxford Metrics, Yarnton, UK) and four KISTLER force plates (Kistler Holding AG, Winterthur, Switzerland). The subject was asked to walk at comfortable speed down the 10 m walkway. Data was collected over three successful trials. The Gait Deviation Index (GDI) was calculated, and the mean value of both lower limbs was used for the statistical analysis [22]. The GDI was selected as a standardized measure of overall kinematic gait deviation; it has demonstrated good reliability and validity in children with CP [23].

The 10-Meter Walk test was used to measure walking speed. Participants were instructed to walk as quickly as possible without running along a 10 m marked walkway. The time taken to complete the distance was recorded [24].

#### 2.5.2. Activity Performance and Participation Assessments

The GMFM-66 was used to evaluate the children’s gross motor function. Only the D (standing) and E (walking, running and jumping) items were evaluated.

Actimetric recording was performed using a three-axis Actigraph^®^ accelerometer (ActiGraph, LLC, Pensacola, FL, USA). Acceleration data from all participants were collected with a 100 Hz sampling frequency. The Actigraph^®^ output (counts per minute) was converted into physical activity intensity units using a specific algorithm [25]. The parameters used in this study were the percentages of time spent in sedentary and moderate-to-vigorous physical activity (MVPA), and numbers of steps per day. Freedson’s children equation was applied to interpret the accelerometer data, using Actilife^®^ software (version 6.13.4). The accelerometer was worn by children around the waist for seven days [26]. During this period, parents or children completed a daily diary, recording all activities and sports performed on an hourly basis. Wear-time validation was conducted using Actilife^®^ software (version 6.13.4) followed by a manual review to ensure accurate wear-time validation.

Quality of life was assessed using the Pediatric Quality of Life Inventory (PedsQL), which evaluates four dimensions: Physical Functioning, Emotional Functioning, Social Functioning, and School Functioning. Each item on the questionnaire offers five response options, with scores ranging from 0 (never a problem) to 4 (almost always a problem) [27]. Both the children and their parents completed the questionnaire.

### 2.6. Intervention

The Activ’Marche program was provided by physical therapists and physical educators 5 times per week for 4 weeks, followed by 2 weeks of home-based telerehabilitation. Training parameters followed the FITT scheme (frequency, intensity, time, and type) and were based on the existing literature. The program combined three types of training: functional gait training, strength training, and HIIT training. HIIT sessions alternated between muscle-strengthening and locomotor formats to increase training dose (Figure 1).

Functional gait training consisted of 40 min overground walking sessions twice per week using habitual assistive devices. Participants walked at a comfortable pace during the first and last five minutes and were encouraged to increase speed during the remaining time [13].

Strength training targeted lower limb muscles using training circuits combining both bodyweight exercises and machine-based resistance exercises. Sessions included a minimum of 5 sets of 10 repetitions per exercise. After an initial familiarization session, training loads were individualized using estimated one-repetition maximum (1-RM). Exercises were performed at 70% 1RM (5 × 10 repetitions) during the first two weeks and at 80% 1-RM (5 × 8 repetitions) during the final two weeks [28].

HIIT included circuit-based strengthening and locomotor sprint training, adapted from Zwinkles et al. [18]. HIIT circuit training comprised an 8 min warm-up followed by three blocks of six intervals (30 s work/60 s rest), with 2 min recovery between blocks. HIIT locomotor training followed the same structure but used 30 s work/30 s rest.

Representative examples of exercises included in the Activ’Marche program, as well as the specialized equipment used during the training sessions, are presented in Figure 2.

Complementary activities (stretching, adapted physical activity, and therapeutic education) were offered during free time to promote long-term physical activity.

Stretching sessions focused on maintaining muscle flexibility and joint range of motion, particularly in the lower limbs. Adapted physical activities included recreational and sport-oriented activities selected according to the participants’ functional abilities and interests, with the aim of encouraging active participation and helping participants identify physical activities that could be continued after discharge.

Therapeutic education sessions were organized twice a week in sub-groups of four patients and addressed key topics such as: What is cerebral palsy? Why did we undergo surgery? Sports and physical activity: which type of sport is right for me?

After inpatient rehabilitation, participants completed two weeks of home-based telerehabilitation, including three supervised strengthening sessions via videoconference and three independent walking sessions per week, combining HIIT locomotor training and functional walking exercises.

### 2.7. Sample Size Calculation

Sample size was calculated based on the primary outcome measure: the 6MWT. Preliminary data from 8 patients (12–18 months post-SEMLS) showed a mean distance of 467.5 m (SD = 84.99). Expected improvement was derived from previous gait training studies in children with CP, which reported gains of 96.11 m and 35.77 m on the 6MWT [13,14]. Assuming a target improvement of 65.94 m, a mean of 467.5 m (SD = 84.99), a power of 0.9, and the Student’s t-test, a sample size of 20 patients was required. Considering potential dropouts, 24 patients were recruited.

### 2.8. Statistical Analysis

Statistical analyses were performed using Matlab version 23.2.0.2459199 (R2023b) Update 5 (The MathWorks, Inc., Natick, MA, USA). For each variable, means and standard deviations or median and interquartile ranges (IQRs) were calculated and comparisons between pre- and post-treatment measurements were performed using parametric tests (paired *t*-tests) or nonparametric tests (Wilcoxon signed-rank tests), depending on the data distribution measured by Shapiro–Wilk normality tests. A *p*-value < 0.05 was considered significant.

## 3. Results

Twenty-three children with CP participated in the Activ’Marche program (Table 1). All participants completed the 4-week intervention, and 19 of 23 followed live telerehabilitation sessions, while the remaining participants received exercise instructions and materials via a USB drive.

### 3.1. Primary Outcome: 6MWT

The 6MWT showed significant improvement across the three assessment points (Table 2, Figure 3a). The mean distance walked increased from 409.3 ± 111.3 m at baseline (Pre) to 447.0 ± 89 m immediately after the intervention (Post), and further to 464.2 ± 83.3 m at the follow-up (PostFinal).

### 3.2. Secondary Outcome:

#### 3.2.1. Gait Deviation Index

The GDI remained relatively stable across the three assessment points. No statistically significant changes were observed between any time points.

#### 3.2.2. GMFM-66 D and E

GMFM-66 D showed significant improvement following the Activ’Marche program (Table 2, Figure 3c). Statistical comparisons indicated a significant improvement from Pre to Post (*p* = 0.007) and from Pre to PostFinal (*p* < 0.001). Additionally, a significant improvement was observed between Post and PostFinal (*p* = 0.004), indicating sustained and progressive gains.

Similarly, GMFM-66 E improved significantly from Pre to Post (*p* < 0.001) and from Pre to PostFinal (*p* < 0.001). A smaller but still significant improvement was noted between Post and PostFinal (*p* = 0.035), suggesting continued progression in complex motor tasks (Table 2, Figure 3d).

#### 3.2.3. 10-Meter Walk Test

The 10MWT showed significant improvements in walking speed. Median time to complete the test decreased from 7.4 s (IQR 6.5–7.9) at baseline to 6.8 s (IQR 6.1–6.9) post-intervention, and further to 6.05 s (IQR 5.4–6.7) at follow-up (Table 2, Figure 3b). Time decreased significantly from Pre to Post (*p* = 0.002) and from Pre to PostFinal (*p* = 0.018), while no difference was observed between Post and PostFinal (*p* = 0.350), suggesting a plateau effect after initial gains.

#### 3.2.4. Actimetric Recording

The actimetric data indicated no significant changes in overall physical activity. Sedentary time remained stable (74.2 ± 6.2% at Pre, 79.6 ± 11.4% at Post, and 76.3 ± 8.0% at PostFinal; *p* > 0.18). Time spent in MVPA also remained low across assessments (median 1.3%, 1.1%, and 0.76%, respectively; *p* > 0.25). Similarly, daily step counts did not differ significantly (4185 ± 2157 at Pre, 3758 ± 1817 at Post, and 4513 ± 2157 at PostFinal; *p* > 0.16).

#### 3.2.5. Quality of Life

Quality of life assessed by PedsQL showed no significant changes. Adolescent self-reports PedsQL scores remained stable across time points (median 73.9 at Pre, 75 at Post, and 77.2 at PostFinal; *p* > 0.44). Parent-reported scores increased from Pre to Post but declined slightly at follow-up; however, no comparisons reached statistical significance (all *p* > 0.19).

## 4. Discussion

This study evaluated the effects of an intensive rehabilitation protocol, Activ’Marche, that combined functional gait training, strength training, and high-intensity interval training one year after single-event multilevel surgery in adolescents with cerebral palsy. The findings support the effectiveness of this approach in improving functional motor outcomes, particularly in terms of walking capacity, standing ability, and overall gross motor function. However, no improvements were observed in GDI, quality of life, or actimetric recordings.

### 4.1. Functional Gains and Clinical Implications

Significant improvements were observed in the 6MWT (+13%), GMFM dimensions D (+6.7%) and E (+8.5%), and the 10MWT (+14,2%), indicating enhancements in endurance, gross motor function, and walking speed. These effect sizes are consistent with or greater than those reported in prior studies using strength training, functional gait training, or HIIT training.

For instance, studies focusing on strength training, such as those by Ryan et al. [29] and Taylor et al. [30], typically reported GMFM improvements in the range of 6–10%, while Dodd et al. [31] highlighted limitations in transferring strength gains to functional performance. Activ’Marche produced GMFM gains comparable to the upper range of the results reported in the literature.

In addition, the effects were even stronger compared to studies using HIIT training or circuit-based aerobic protocols, such as Swe et al. [14] and Cho et al. [12], who reported around 6–10% improvements in walking capacity.

Regarding functional gait training, Fosdahl et al. [32] and Schranz et al. [33] documented improvements of 6–9% in the 6MWT and GMFM scores, which are slightly below the levels seen in this study.

The continued improvement seen at the three-month follow-up further supports the idea that such training not only enhances capacity in the short term but may also promote a prolonged improvement in physical capacities.

These comparisons should nevertheless be interpreted cautiously, as differences in population characteristics, age and postoperative context limit direct comparisons. Thus, the present findings should not be interpreted as evidence of superiority of Activ’Marche over other training protocols.

### 4.2. Gait Deviation Index and Quality of Life

Interestingly, despite gains in function and especially in walking speed, no significant change was observed in the GDI, which quantifies the kinematic gait pattern deviation. This outcome aligns with our expectations and initial hypothesis. Indeed, gait quality often improves rapidly after surgery and conventional rehabilitation, which already addresses many biomechanical deficits. The goal of this study was precisely to enhance motor parameters related to gait functionality and functional gains. Therefore, the lack of further improvement in GDI may reflect a ceiling effect in biomechanical quality, particularly given that the neuromuscular and skeletal changes targeted by SEMLS may impose limitations that are difficult to manage through training alone, especially in individuals more than one year post-surgery.

Furthermore, the absence of change in GDI is an important finding, as it indicates that Activ’Marche did not have any detrimental effect on gait quality. The stability of GDI throughout the program suggests that such targeted functional training can be safely implemented without compromising gait quality.

Similarly, no significant improvements were found in PedsQL scores, despite the improvements in physical function. This disconnection between motor capacity and perceived quality of life may be due to several factors, including psychosocial challenges, environmental limitations, or the relatively short timeframe of the intervention. Furthermore, expectations for improvement might be higher among adolescents, and the perceived burden of intensive rehabilitation could offset positive physical gains in their self-assessments.

### 4.3. Activity Performance Outcomes and Participation: A Complex Challenge

Contrary to expectations, actimetric recordings did not improve significantly. While there was a trend toward increased step count at follow-up, this did not reach statistical significance. These findings highlight the complexity of translating gains in physical capacity into increased real-world activity.

However, the primary aim of the Activ’Marche protocol was mainly to target functional recovery and walking capacity in the late postoperative period. The relatively short duration of home-based rehabilitation may have been insufficient to produce measurable behavioral changes in daily activity.

Future programs could benefit from the integration of therapeutic education components, greater family involvement, and long-term community-based initiatives that promote physical activity among youth with cerebral palsy [34,35].

### 4.4. Strengths and Limitations

The Activ’Marche protocol was co-developed with an experienced multidisciplinary team of therapists. This new intensive rehabilitation stage combining inpatient and telerehabilitation addresses the same global goals targeted by the fourth step of general post-operative protocols, while enhancing rehabilitation intensity. It evaluates the effect of a combined, multidisciplinary intervention which integrates functional gait training, strength training, and HIIT, unlike many studies in the literature that typically focus on a single intervention approach.

However, this study has some limitations. First, the sample size and the absence of a control group limit the generalizability of the findings and the ability to draw definitive conclusions about the causal effects of the intervention or about any superiority over other comparable protocols. Second, the limited improvements observed in participation and quality of life suggest that physical training alone may not be sufficient to drive broader psychosocial benefits. This highlights the potential need for complementary behavioral and social support strategies.

## 5. Conclusions

The Activ’Marche intensive rehabilitation protocol, introduced one year after SEMLS, may improve functional walking capacity and gross motor performance in adolescents with CP. Our findings indicate meaningful improvements in walking speed and gross motor function, with some gains continuing beyond the end of the intensive intervention.

However, no significant changes were observed in gait kinematics, daily physical activity, or quality of life, suggesting that improvements in physical capacity may not necessarily translate into broader participation outcomes.

Overall, these findings support the potential value of a late intensive, multimodal rehabilitation phase after SEMLS, while further controlled studies with larger samples are needed to confirm its effectiveness and determine its specific contribution to long-term functional outcomes.

## Figures and Tables

**Figure 1:**
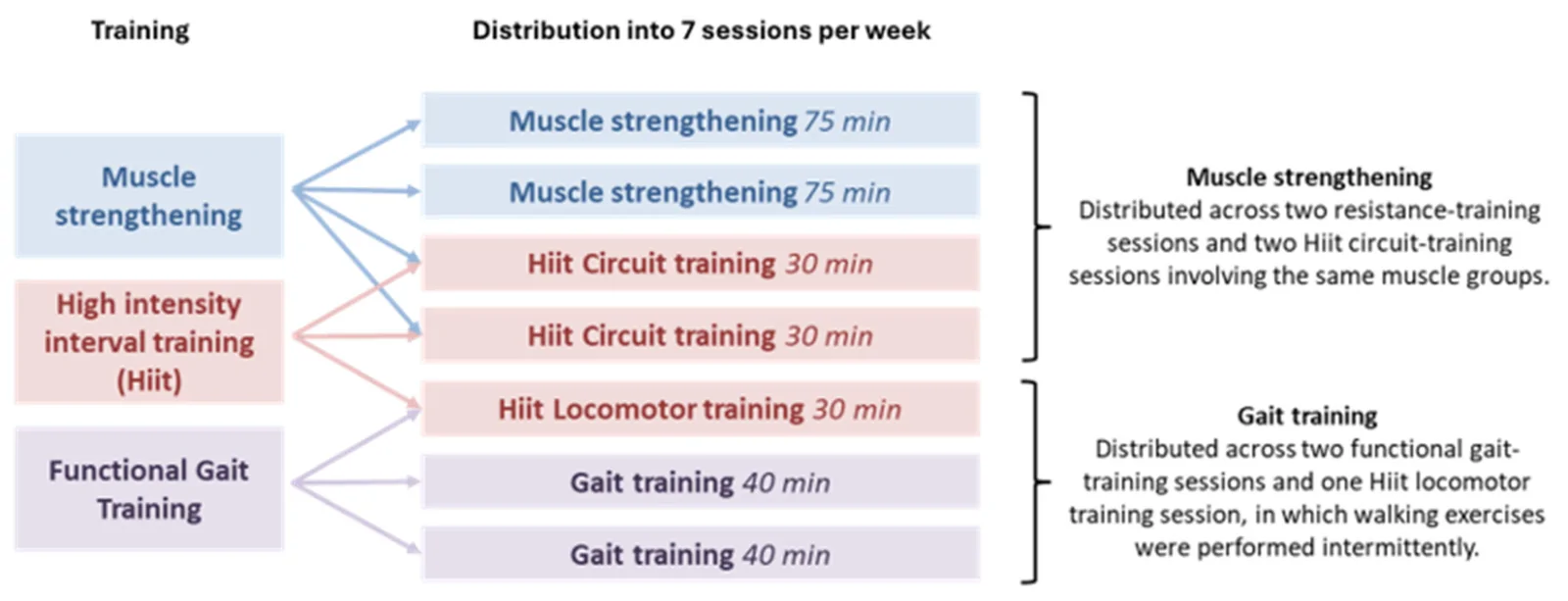
Overview of training types, duration and distribution in the Activ’Marche program.

**Figure 2:**
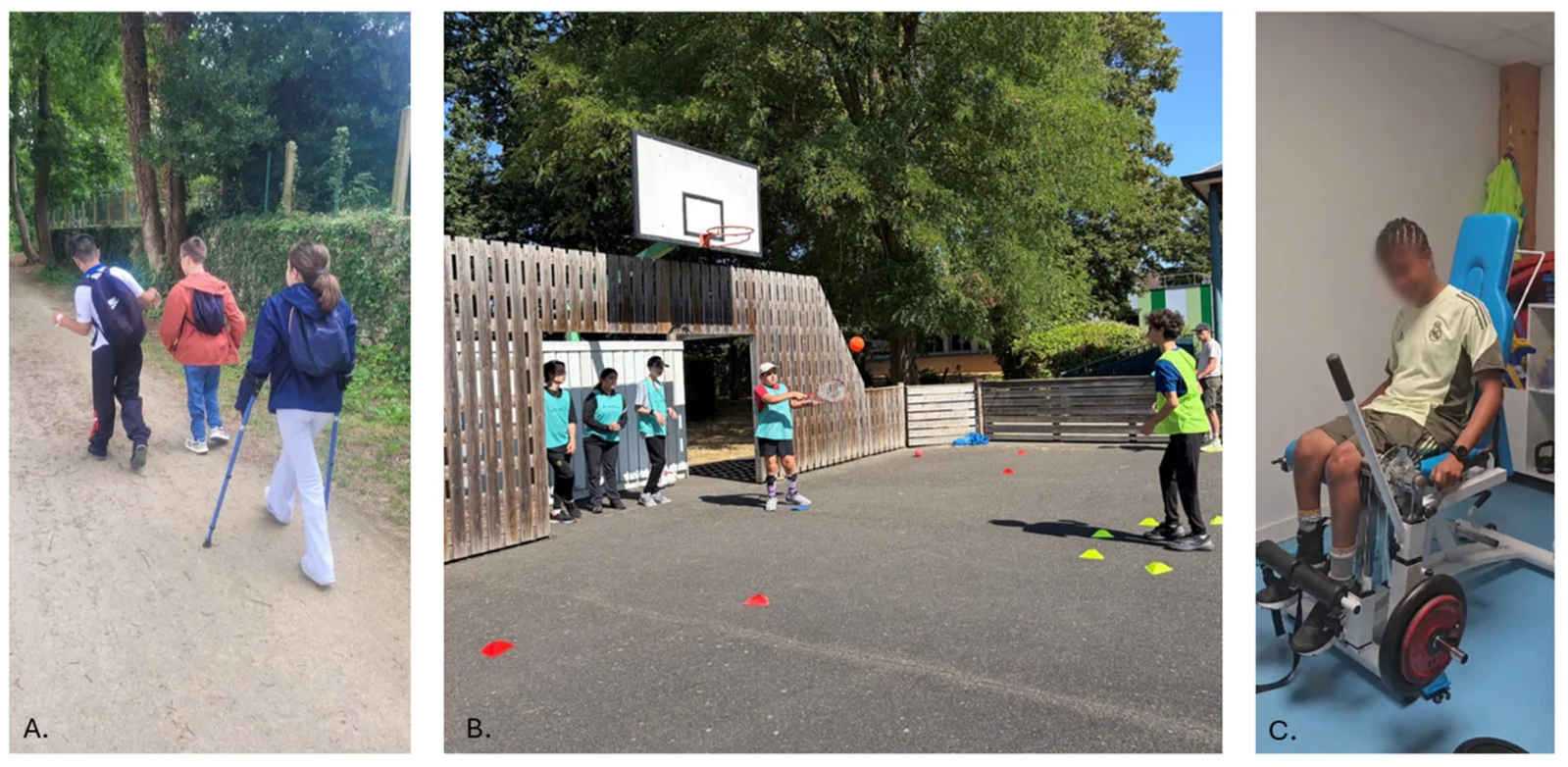
Representative examples of exercises included in the Activ’Marche program: (**A**) functional gait training, (**B**) HIIT locomotor training and (**C**) strength training.

**Figure 3:**
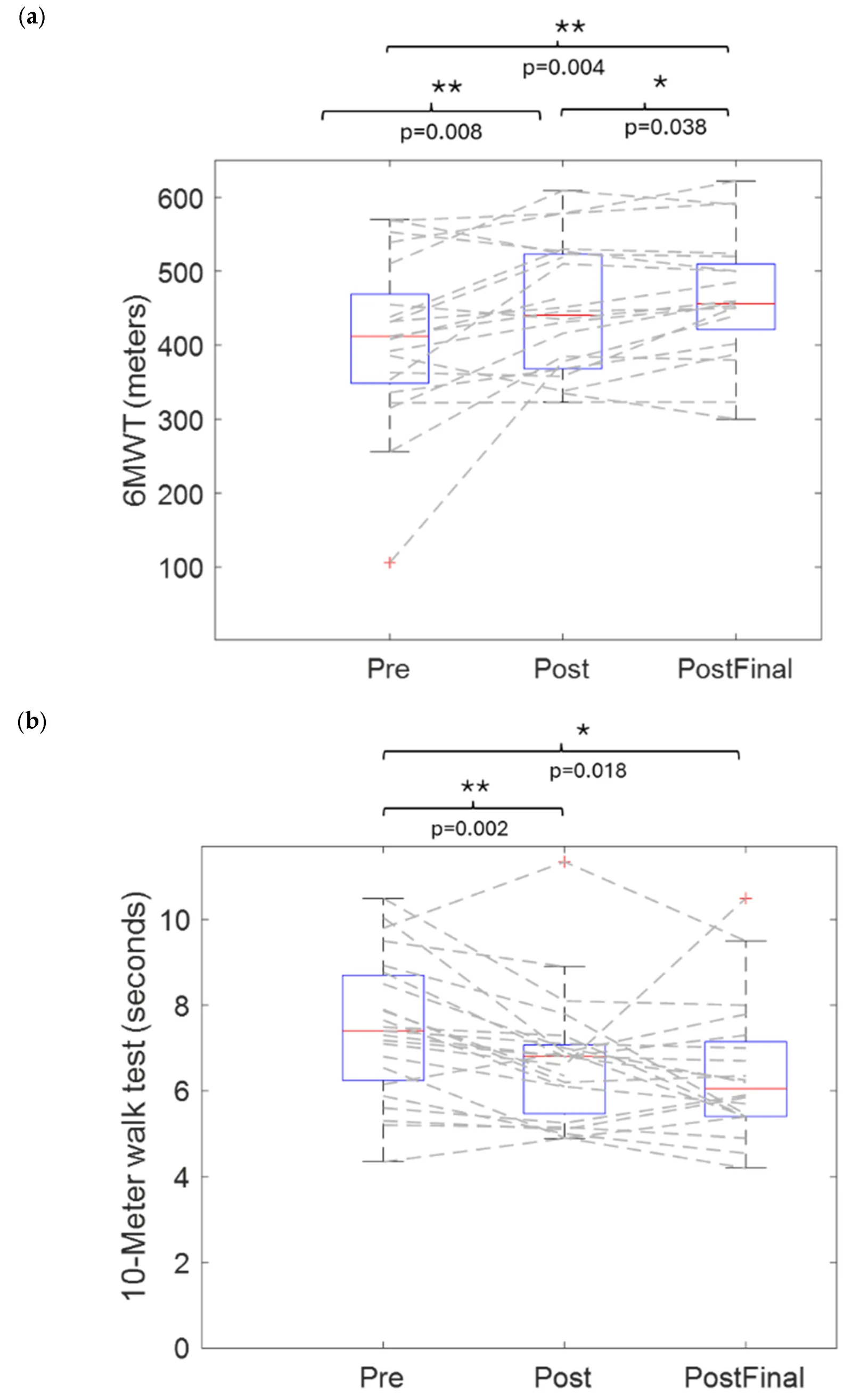

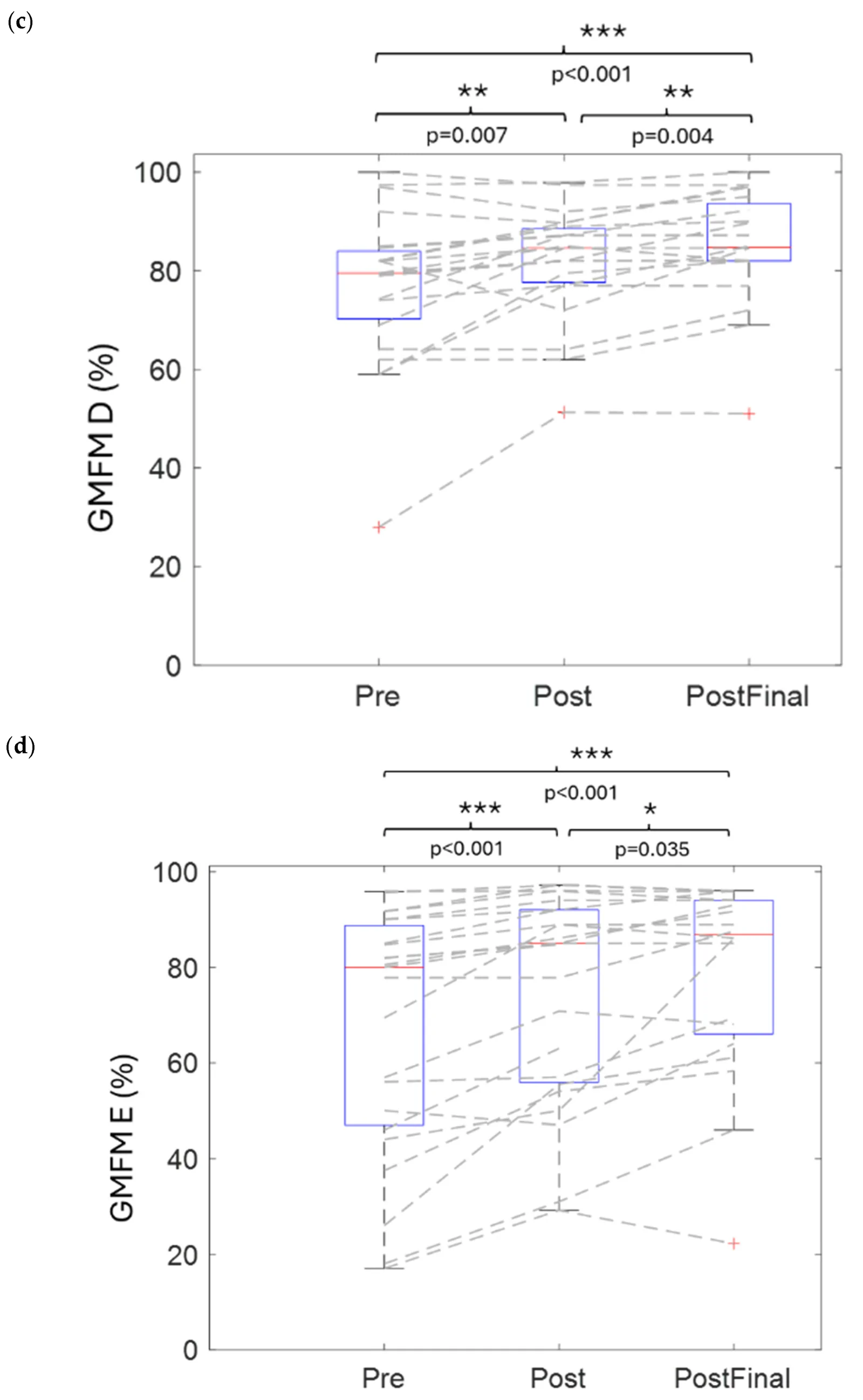
Box plots presenting the values of (**a**) 6MWT, (**b**) 10MWT, (**c**) GMFM D and (**d**) GMFM E during the different assessment stages of Activ’Marche intensive therapy.The red horizontal line indicates the median value, and the blue boxes represent the interquartile range (IQR; 25th and 75th percentiles, and maximum and minimum values. For each subject, the dashed lines link the Pre, Post and Postfinal values. * *p* < 0.05; ** *p* < 0.01; *** *p* < 0.001.

**Table 1:** Descriptive characteristics of the participants according to the Gross Motor Classification Scale (GMFCS).

	Overall Mean (SD) N = 23	GMFCS I Mean (SD) N = 14	GMFCSII Mean (SD) N = 9
**Age**	15.7 (1.9)	16.3 (1.9)	14.8 (1.7)
**EFMG-D**	76.9 (15.6)	81.6 (17.3)	69.7 (9.2)
**EFMG-E**	67.3 (25.6)	78.2 (22.1)	50.3 (21.8)
**6 MWT**	409.3 (111.2)	441.6 (123.3)	356.7 (65)
**GDI**	75.9 (6.3)	78.6 (6.2)	71.8 (4)
**Steps per day**	4184.7 (2156.5)	4733.4 (2328.3)	3087.4 (1284.3)
**% in Sedentary**	74.2 (6.2)	72.6 (5.3)	77.2 (7)
**% in MVPA**	1.8 (1.9)	2.3 (2.1)	0.7 (0.6)

**Table 2:** Outcomes across the three assessment points.

Outcome	Baseline (Pre)	Post-Intervention (Post)	Follow-Up (PostFinal)	Time Effect (*p*-Value)
**Primary Outcome**				
**6MWT (mean ± SD)**	409.3 ± 111.3	447.0 ± 89	464.2 ± 83.3	Pre vs. Post: 0.008 Pre vs. PostFinal: 0.004 Post vs. PostFinal: 0.038
**Secondary Outcomes**				
**Gait Deviation Index (median, IQR)**	76.2 (71–80.5)	73.3 (68.6–78.4)	73.5 (70–80.7)	NS
**GMFM-66 Dimension D (median, IQR)**	79.5 (74–82)	84.6 (79.5–87.2)	84.8 (82–90)	Pre vs. Post: 0.007 Pre vs. PostFinal: <0.001 Post vs. PostFinal: 0.004
**GMFM-66 Dimension E (median, IQR)**	80.0 (50–84.7)	85.0 (57–88.9)	86.8 (68–93)	Pre vs. Post: <0.001 Pre vs. PostFinal: <0.001 Post vs. PostFinal: 0.035
**10-Meter Walk Test (seconds, median, IQR)**	7.4 (6.5–7.9)	6.8 (6.1–6.9)	6.05 (5.4–6.7)	Pre vs. Post: 0.002 Pre vs. PostFinal: 0.018 Post vs. PostFinal: 0.350
**Actimetric Recording**				
**% Sedentary Time (mean ± SD)**	74.2 ± 6.2	79.6 ± 11.4	76.25 ± 8.03	NS
**% MVPA (median, IQR)**	1.3 (0.2–1.9)	1.1 (0.1–3.4)	0.76 (0.37–1.47)	NS
**Steps/Day (mean ± SD)**	4184.7 ± 2156.5	3757.6 ± 1817.4	4513.2 ± 2156.6	NS
**Quality of Life (PedsQL)**				
**Adolescent Self-Report (median, IQR)**	73.9 (67–79.3)	75 (68.5–80)	77.2 (65.2–84.8)	NS
**Parent Report (mean ± SD)**	63.69 ± 12.25	70.67 ± 13.79	67.83 ± 16.84	NS

## Data Availability

Available from the corresponding author upon request.

## Abbreviations

The following abbreviations are used in this manuscript:

CP	Cerebral Palsy
GMFCS	Gross Motor Function Classification System
GMFM-66	Gross Motor Function Measure-66
GDI	Gait Deviation Index
HIIT	High-Intensity Interval Training
ICF-CY	International Classification of Functioning, Disability and Health: Children & Youth version
MVPA	Moderate-to-Vigorous Physical Activity
SEMLS	Single-Event Multilevel Surgery
PedsQL	Pediatric Quality of Life Inventory
1-RM	Maximum One-Repetition
6MWT	6-Minute Walk Test
10MWT	10-Meter Walk Test
